# Indirect Genetic Effects for Growth Rate in Domestic Pigs Alter Aggressive and Manipulative Biting Behaviour

**DOI:** 10.1007/s10519-014-9671-9

**Published:** 2014-09-17

**Authors:** Irene Camerlink, Winanda W. Ursinus, Piter Bijma, Bas Kemp, J. Elizabeth Bolhuis

**Affiliations:** 1 Department of Animal Sciences, Adaptation Physiology Group, Wageningen University, PO Box 338, 6700 AH Wageningen, The Netherlands; 2 Department of Animal Sciences, Animal Breeding and Genomics Centre, Wageningen University, PO Box 338, 6700 AH Wageningen, The Netherlands; 3 Animal Behaviour and Welfare, Wageningen UR Livestock Research, PO Box 65, 8200 AB Lelystad, The Netherlands

**Keywords:** Behaviour, Indirect genetic effects, Genotype-environment interaction, Pig, Tail biting, Response to selection

## Abstract

Indirect genetic effects (IGEs) are heritable effects of an individual on phenotypic values of others, and may result from social interactions. We determined the behavioural consequences of selection for IGEs for growth (IGEg) in pigs in a G × E treatment design. Pigs (*n* = 480) were selected for high versus low IGEg with a contrast of 14 g average daily gain and were housed in either barren or straw-enriched pens (*n* = 80). High IGEg pigs showed from 8 to 23 weeks age 40 % less aggressive biting (*P* = 0.006), 27 % less ear biting (*P* = 0.03), and 40 % less biting on enrichment material (*P* = 0.005). High IGEg pigs had a lower tail damage score (high 2.0; low 2.2; *P* = 0.004), and consumed 30 % less jute sacks (*P* = 0.002). Selection on high IGEg reduced biting behaviours additive to the, generally much larger, effects of straw-bedding (*P* < 0.01), with no G × E interactions. These results show opportunities to reduce harmful biting behaviours in pigs.

## Introduction

Social interactions among individuals may affect a variety of phenotypic traits (e.g. Frank 2007). If these social effects on others are heritable they may affect response to selection, and thereby alter the outcome of both evolutionary processes in natural populations, and artificial selection programs in agriculture (e.g. Griffing 1967; Bijma and Wade 2008; McGlothlin et al. 2010). The impact of social interactions on response to selection can be studied within the framework of indirect genetic effects (IGEs). An indirect genetic effect (IGE), also known as an associative, social-, or competitive genetic effect, or a social breeding value, is a heritable effect of an individual on the trait values of its social partners (Griffing 1967; Moore et al. 1997). For example, an individual may reduce the growth of its social partners because it carries genes making it highly competitive. IGEs are relevant both for the evolution of natural populations, and for response to artificial selection in domestic and agricultural populations, ranging from trees to laboratory animals and livestock (Wolf et al. 1998; Bijma 2011). Theory predicts that IGEs affect the response to selection (Griffing 1967; Moore et al. 1997; Bijma et al. 2007), and there is a growing body of evidence for the existence of IGEs (e.g. Peeters et al. 2012; Alemu et al. 2014). Studies indicate that competitive, aggressive, or injurious behaviours, but also cooperation, may underlie the observed IGEs (Agrawal et al. 2001; Mutic and Wolf 2007; Wilson et al. 2009; Rodenburg et al. 2010; Alemu et al. 2014). The link between IGEs and behaviour is especially relevant to livestock populations, where behaviour is an important component of animal welfare. First selection experiments in poultry yielded promising results on production and behaviour (e.g. Muir 1996; Rodenburg et al. 2010; Muir et al. 2013), and revealed changes in the neuroendocrine system of laying hens (reviewed in Cheng 2010). Yet, animal scientists are only at the start of discovering mechanisms underlying IGEs, and there is an urge for more empirical research (Wilson 2013).

In domestic pigs (*Sus scrofa*), IGEs affect growth rate (here denoted as IGEg), meaning that pigs differ in the heritable effect they express on the growth rate of their pen mates (e.g. Bergsma et al. 2013). Commercially kept pigs have been selected primarily for growth rate and are kept in barren environments, which both may have increased competitive and aberrant behaviour (Rodenburg and Turner 2012). Aberrant behaviour, such as repeatedly chewing on tails or ears of group mates is in some pig breeds heritable (Breuer et al. 2005), may harm growth and health of the bitten animal, and is considered a severe welfare problem in pig husbandry (e.g. Schrøder-Petersen and Simonsen 2001). Selection on IGEg might contribute to a solution to simultaneously improve both productivity and welfare (Rodenburg et al. 2010).

Consequences of selection for IGEg on the behavioural repertoire of pigs are largely unknown, as well as the potential dependency of IGEg on the environment. The genetic disposition for certain behaviours, for example aggression, may be expressed differently depending on the environment (e.g. Barr et al. 2003). It is therefore important to consider genotype-environment interactions (G × E) to assess whether changes due to selection for IGEs are consistent across environments (Danielson-François et al. 2009).

The objective was to study whether selection for IGEs for growth (IGEg) alters the behaviour of pigs, and whether interactions exist between IGEg and the environment regarding behaviour. This was investigated in a one generation selection experiment whereby pigs were divergently selected for IGEg, and housed in contrasting conditions (barren versus straw-enriched) that were expected to yield differences in behaviour. This is one of the first selection experiments on IGEs in a large mammal. The results will provide insight in the mechanisms underlying IGEs for growth, and in the potential of selection on IGEs to improve social interactions between group living animals.

## Materials and Methods

### Genetic Selection on IGE for Growth (IGEg)

Background information on IGEs, and the estimation of IGEs for growth during the finishing phase (from 25 to 110 kg) for the current trial, here denoted as **IGEg**, has been given in detail in Camerlink et al. (2013). Briefly, sows (64 Topigs-20 sows: sow line of Great Yorkshire × Dutch Landrace) and boars (24 Tempo boars: commercial synthetic boar line with Great Yorkshire genetic background) were selected based on their estimated breeding value for IGEg. Sires and dams with the most extreme high and low IGEg of the available population were mated within their IGEg group (high vs. low), while the direct breeding value was kept equal between groups. This resulted in a contrast of 2.8 g ADG (average daily gain) between high and low IGEg offspring (40 high IGEg litters and 40 low IGEg litters). With 6 pigs per pen this results in a total contrast of 14 g ADG, i.e. (6−1) × 2.8 = 14. Hence, high IGEg offspring would increase the growth of their pen mates, whereas low IGEg offspring would decrease the growth of their pen mates (effects on growth have been reported in Camerlink et al. 2014). Offspring were studied over five batches of 96 pigs each (*n* = 480), between September 2010 and February 2012.

### Animals and Housing

Piglets were born in conventional farrowing pens with farrowing crates (TOPIGS experimental farm, Beilen, The Netherlands). Tails and teeth were kept intact. Male piglets were castrated (at 3 days of age) because IGEg have currently been estimated on gilts and castrated males. Cross fostering was applied only if litter sizes exceeded 14 piglets, and always within the same IGEg group. At ~14 days of age, piglets were subjected to the backtest to assess their coping style (Hessing et al. 1993). Classification of piglets based on their response in the backtest, for which no relationship with IGEg was found (Reimert et al. 2013), was used to standardize group composition with regard to coping style. Piglets were weaned at 26 days of age, whereby maximum eight piglets per sow were selected. Selection was based on good health, sex (1:1), and backtest response (to the ratio of the tested population). Selected piglets (*n* = 480 in total) were transported to experimental farm De Haar (Wageningen, The Netherlands).

From weaning to slaughter (4–23 week of age), a 2 × 2 experimental arrangement was applied with IGEg (low vs. high) and housing conditions (barren vs. enriched) as factors at the pen level. Pigs were housed with six per pen, leading to 80 pens in total. Group composition was balanced for sex (1:1) and backtest classification (at least two of each classification). Half of the pigs from each IGEg group, and half of the selected piglets from each sow, were allocated to barren pens and the other half to enriched pens. Pens were composed of pigs which were unfamiliar to each other, i.e. no littermates or sibs were kept together.

Barren pens had a floor which was half solid concrete and half slatted. Enriched pens had a solid floor with a bedding of 12 kg of wood shavings and 1.5 kg of straw. Fresh wood shavings (3 kg/pen) and straw (0.25–1.5 kg/pen depending on age) were added to enriched pens daily. Pen dimensions were either 1.90 × 3.20 m or 2.25 × 3.25 m (1–1.2 m^2^/pig), depending on batch, and were within batch equal between barren and enriched pens. All pens had a metal chain with ball attached to the pen wall as toy. Dry pelleted commercial feed was offered ad libitum from a single space feeder. Feed was provided according to commercial practice, with a total of four feed changes whereby on the first day the old and new feed types were mixed to create a gradual transition between feed types. Water was continuously available from a single nipple drinker per pen. Temperature was until 10 days after weaning set at a minimum of 25 °C, and was hereafter set at 22 °C for 3 weeks, followed by 20 °C until slaughter. Lights and a radio were on from 7:00 till 19:00 h. To reduce damaging tail biting behaviour, i.e. chewing on the tail of a conspecific which can lead to injury and in extreme cases even to mortality of the bitten animal, all pens received a handful of wood shavings per day from week 6 onwards and a jute sack was attached to the wall from week 8 onwards. Pigs were housed in these pens from weaning until slaughter. Due to diverse health reasons including tail biting, 18 high IGEg and 11 low IGEg pigs were removed from the experiment.

### Behavioural Observations

Behaviours of individual pigs were recorded at 4, 5, 8, 12, 16, and 21 weeks of age. Each pig was identified by a spray marked number on the back, which was refreshed before behavioural observations. Behaviour, as described in Table 3 (Appendix), was scored during live observations using 2-min instantaneous scan sampling for 6 h during the active period of the day, consisting of six 1 h blocks from 8:00 to 11.30 h and from 14.00 to 17:30 h with after each hour a 15 min break. This procedure resulted in 180 observations per pig per observation day, with one observation day in each of the weeks mentioned. The Observer 5.0 software package (Noldus Information Technology B.V., Wageningen, The Netherlands) installed on a hand-held computer was used for behaviour recordings. Observations were carried out by observers who were unaware of the IGEg of the pigs.

### Tail Damage Scores

Tail damage scores can serve as an indicator for the amount of tail biting behaviour in a pen. Scores were obtained using an adapted procedure from Zonderland et al. (2008). Scores ranged from 1 to 4, with score 1 being no visible tail damage; score 2 for hair removed from the tail; score 3 for bite marks; and score 4 for a clearly visible wound. Tail damage was scored each week on each individual pig, leading up to 20 observations per pig. When a pig had to be removed from the trial due to being bitten severely its score was set to 4 for the remaining period till slaughter. When a tail biter had to be removed from the pen it kept its last score before being removed from the pen. Scores were obtained by multiple observers who were trained to score in the same way, and who were unaware of the IGEg of the pigs.

### Interventions to Limit Damage Due to Tail Biting

**Fig. 1:**
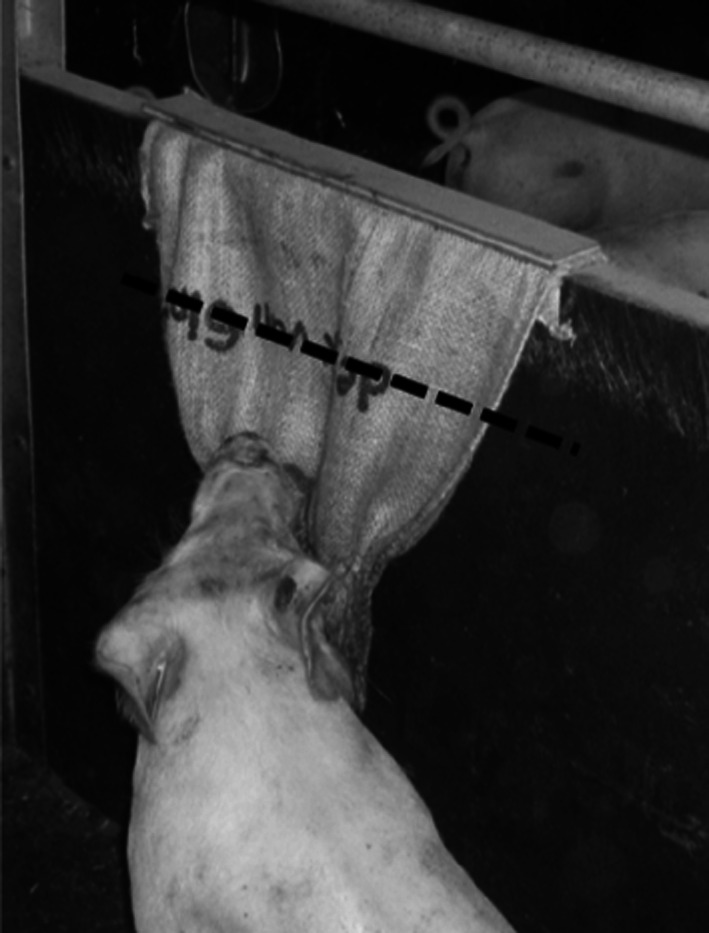
Jute sack attached to pen wall as distraction material to limit tail biting. The sack was replaced when the sack was ‘consumed’ till the *dashed line* or further

Oral manipulation amongst pigs is the repeatedly biting on the tail, ear or paw of a group member, and may result in injury, impaired health or mortality of the bitten animal. Oral manipulation such as tail biting may start harmlessly, but when no measures are taken many animals may be severely damaged (Statham et al. 2009). During the trial, measures were taken to reduce tail biting to an acceptable level to prevent the loss of animals and to guarantee a certain level of animal welfare. Tail biting wounds became significant from 6 weeks of age. To reduce the amount of damaging tail biting behaviour, a handful of wood shavings was provided to each pen from week 6 onward and from week 8 a jute sack was attached to the pen wall as material to chew on. The jute sack was a commercially available sack of approximately 60 × 105 cm, which was over the width attached to the pen wall and was replaced when there was less than 1/3 of the sack left (Fig. 1). When the sack was replaced, the remainders were approximated in cm^2^. The amount of jute sack that was ‘consumed’ was noted by pen. To reduce tail biting, the tails of bitten pigs were alternating between days covered with the aversive P.B.H. spray (Kommer Biopharm B.V.) or Stockholm tar (Rapide^®^). Pigs were removed from the pen when they had a reduction in tail length, irrespective of the amount of reduction. Six high IGEg pigs and three low IGE pigs, from eight different pens in total, were removed from the trial due to reduced tail length. One tail biter (low IGEg) was removed to limit further tail damage of its five pen mates.

### Data Analyses

Statistical analyses were performed using SAS (SAS 9.2, Institute Inc.). Data were analysed and presented by production phase as applied in commercial pig farming to facilitate comparison between animal behaviour studies. The nursery phase is from 4 to 8 weeks of age, whereas the finishing phase is generally from 8 weeks of age till slaughter (here at 23 weeks of age).

Behavioural scans were analysed on pen level (*n* = 80) and averaged over production phase (nursery phase: observations weeks 4, 5, and 8; finishing phase: observations weeks 11, 12, 16 and 21). Hereto the behaviours of pigs were averaged by pen (6 pigs/pen). Residuals of the response variables were checked for normality, and if needed, behaviours were arcsine square root transformed. Behaviours by pen and production phase were analysed in a general linear model (GLM procedure), and included IGEg group, housing condition, the interaction between IGEg group and housing condition, and batch as fixed class effects.

The weekly tail damage scores were averaged into two scores per pig, one for the nursery phase (weeks 4–7) and one for the finishing phase (weeks 8–23). Scores were analysed at individual animal level (*n* = 480) in a generalized linear mixed model (MIXED procedure) with IGEg group, housing condition, the interaction between IGEg group and housing condition, sex, and batch as fixed class effects, and as random factor pen nested within IGEg group, housing condition and batch.

The total cm^2^ of ‘consumed’ jute sacks per pen (from weeks 8–23) was analysed at pen level (*n* = 80) in a general linear model (GLM procedure) with IGE group, housing condition, the interaction between IGE group and housing condition, and batch as fixed class effects. To facilitate the interpretation of consumed bags in cm^2^, results are presented in number of jute sacks consumed [total cm^2^/(60 × 105)]. The amount of jute sacks per pen was correlated to the average tail damage scores per pen by Pearson correlation.

In the results, average trait values for the treatments are reported as (untransformed) LSmeans ± SEM. *P* values below 0.05 are considered significant.

## Results

### Nursery Phase

Over the observation moments between weeks 4 and 8 of age, differences in behaviour between the IGEg groups were small, and did not show a systematic pattern. Pigs with high IGEg showed 20 % less nose contact with pen mates (nose–nose and nose–body contact), and tended to show 25 % less aggressive biting (Table 1). In addition, high IGEg pigs tended to spent less time lying inactive and defecate less than low IGEg pigs (Table 1). There was no difference in overall activity (all activity minus lying inactive and sleeping) (*P* = 0.54), the sum of all explorative behaviours (see Appendix for behaviours) (*P* = 0.55), or the sum of all aggressive behaviours (*P* = 0.85). IGEg group interacted with housing condition for drinking and belly nosing, and tended to interact for rooting, nose contact, and head knocks (Table 1). Other behaviours were not significantly affected by IGEg group, or its interaction with housing.

**Table 1:** Behaviours during the nursery phase (weeks 4–7) in percentage of behavioural scans for each treatment group: high and low IGEg pigs both in barren (B) and enriched (E) pens (*n* = 80 in total), with *P* values for the difference between IGEg groups (*P*-IGE), the difference between housing conditions (*P*-HC), and their interaction (IGE × HC)

Behav. nursery	High E	High B	Low E	Low B	SEM	*P*-IGE	*P*-HC	IGE × HC
Sleeping	38	47	39	46	1.1	0.94	<0.001	0.30
Lying inactive	9.6	12.8	10.6	13.2	0.39	0.08	<0.001	0.44
Standing	2.2	2.9	2.0	2.7	0.2	0.31	<0.001	0.91
Locomotion	2.5	2.5	2.6	2.7	0.13	0.24	0.89	0.72
Sitting	0.82	0.90	0.78	1.0	0.06	0.60	0.01	0.25
Comfort behav.	0.31	0.24	0.30	0.24	0.03	0.92	0.02	0.87
Eating	7.9	8.2	8.7	8.3	0.2	0.89	0.09	0.56
Drinking	1.5	1.8	1.6	1.5	0.09	0.26	0.22	0.02
Urinate/defecate	0.29	0.45	0.36	0.5	0.03	0.09	<0.001	0.75
Playing	1.1	0.63	0.96	0.66	0.1	0.66	<0.001	0.46
Exploration floor	16	11	14	11	0.5	0.14	<0.001	0.43
Nosing object	1.8	2.5	1.9	2.9	0.14	0.13	<0.001	0.37
Rooting	5.8	1.6	5.4	2.2	0.4	0.49	<0.001	0.10
Rooting object	0.07	0.26	0.09	0.31	0.03	0.23	<0.001	0.59
Chewing	10	3	10	3	0.5	0.73	<0.001	0.69
Chewing toy	0.28	0.33	0.15	0.37	0.04	0.26	0.0002	0.13
Nosing body	0.60	1.0	0.63	0.96	0.05	0.87	<0.001	0.55
Nose contact	0.26	0.23	0.27	0.34	0.03	0.03	0.47	0.06
Belly nosing	0.05	0.53	0.11	0.25	0.09	0.20	<0.001	0.02
Mounting	0.26	0.23	0.19	0.26	0.03	0.53	0.53	0.12
Fighting	0.19	0.18	0.15	0.25	0.03	0.18	0.15	0.15
Head knock	0.16	0.11	0.09	0.13	0.02	0.17	0.07	0.07
Biting	0.08	0.10	0.09	0.14	0.01	0.06	0.005	0.23
Fighting at feeder	0.09	0.11	0.08	0.10	0.01	0.54	0.23	0.99
Tail biting	0.02	0.15	0.02	0.17	0.02	0.80	<0.001	0.46
Ear biting	0.10	0.40	0.09	0.37	0.03	0.63	<0.001	0.92
Manip. other	0.11	0.50	0.15	0.50	0.04	0.40	<0.001	0.30

Values are LSmeans of untransformed data with standard error (SEM)

### Finishing Phase

During the finishing phase, when pigs were observed at 12, 16 and 21 weeks of age, high IGEg pigs showed systematically less biting behaviour than low IGEg pigs. Although the frequencies of the observed behaviours are low, of the observed time high IGEg pigs spent 40 % less on aggressive biting of pen mates, and 27 % less on oral manipulation in the form of biting the ears of pen mates than low IGEg pigs did (Table 2). High IGEg pigs were not only biting their pen mates less, but also their environment. They were chewing 40 % less on the distraction materials provided, which were the chain with ball and jute sack (Table 2). High IGEg pigs were 40 % more often observed to perform comfort behaviour, such as scratching the skin (Table 2). Similar to the nursery phase, high IGEg pigs tended to urinate and defecate less than low IGEg pigs (Table 2). There was no difference between the IGEg groups in overall activity (*P* = 0.31), explorative behaviour (*P* = 0.46), or aggressive behaviour (*P* = 0.29). There was a significant interaction between IGEg group and housing condition for lying inactive (*P* = 0.03) and locomotion (*P* = 0.04), see Table 2, and there tended to be G × E interactions for comfort behaviour, drinking, pen exploration, and nosing objects (interactions described in Table 2).

**Table 2:** Behaviours during the finishing phase (weeks 8–23) in percentage of behavioural scans for each treatment group: high and low IGEg pigs both in barren (B) and enriched (E) pens, with *P* values for the difference between IGEg groups (*P*-IGE), the difference between housing conditions (*P*-HC), and their interaction (IGE × HC)

Behav. finishing	High E	High B	Low E	Low B	SEM	*P*-IGE	*P*-HC	IGE × HC
Sleeping	51	55	50	53	1	0.14	0.004	0.54
Lying inactive	14	17	16	17	0.4	0.12	0.002	0.03
Standing	1.1	0.88	0.96	0.95	0.1	0.65	0.12	0.15
Locomotion	0.97	0.79	0.76	0.82	0.1	0.11	0.33	0.04
Sitting	1.9	1.3	2.0	1.4	0.1	0.50	<0.001	0.80
Comfort behav.	0.13	0.07	0.08	0.05	0.01	0.005	<0.001	0.06
Eating	7.2	8.0	7.2	8.1	0.2	0.72	<0.001	0.91
Drinking	2.8	1.9	2.8	2.3	0.1	0.13	<0.001	0.08
Urinate/defecate	0.26	0.34	0.32	0.36	0.03	0.09	0.02	0.41
Playing	0.08	0.05	0.13	0.11	0.03	0.14	0.28	0.69
Exploration floor	8.0	6.0	7.5	6.8	0.4	0.73	0.004	0.09
Nosing object	1.9	1.4	1.8	1.7	0.1	0.37	0.004	0.08
Rooting	1.8	0.4	1.6	0.45	0.1	0.82	<0.001	0.40
Rooting object	0.08	0.06	0.08	0.07	0.01	0.99	0.74	0.85
Chewing	5.8	3.5	5.6	3.4	0.2	0.41	<0.001	0.86
Chewing toy	0.82	1.1	1.1	1.8	0.2	0.005	0.03	0.22
Nosing body	0.75	0.87	0.79	1.0	0.1	0.21	0.02	0.52
Nose contact	0.17	0.17	0.15	0.15	0.02	0.34	0.76	0.95
Belly nosing	0.03	0.13	0.02	0.09	0.03	0.37	0.002	0.40
Mounting	0.03	0.00	0.01	0.01	0.01	0.45	0.18	0.23
Fighting	0.05	0.03	0.03	0.02	0.01	0.25	0.07	0.39
Head knock	0.03	0.04	0.04	0.03	0.01	0.82	0.80	0.92
Biting	0.01	0.04	0.04	0.05	0.01	0.006	0.03	0.30
Fighting at feeder	0.03	0.05	0.05	0.06	0.01	0.30	0.19	0.97
Tail biting	0.05	0.18	0.07	0.17	0.02	0.70	<0.001	0.51
Ear biting	0.08	0.14	0.11	0.18	0.02	0.03	0.004	0.86
Manip. other	0.17	0.40	0.20	0.40	0.04	0.70	<0.001	0.73

Values are LSmeans of untransformed data with standard error (SEM)

### Effect of Housing Condition on Behaviour

Enrichment with straw significantly influenced almost all behaviours during the nursery and finishing phase (Tables 1, 2). Pigs in enriched pens were more active compared to pigs in barren pens, which was seen from less time spent on sleeping, lying inactive and standing. Pigs in enriched pens especially showed less tail biting, ear biting, and belly nosing, and instead spent more time on play, comfort behaviour, and nosing and rooting the pen than pigs in barren pens.

### Tail Damage Scores

**Fig. 2:**
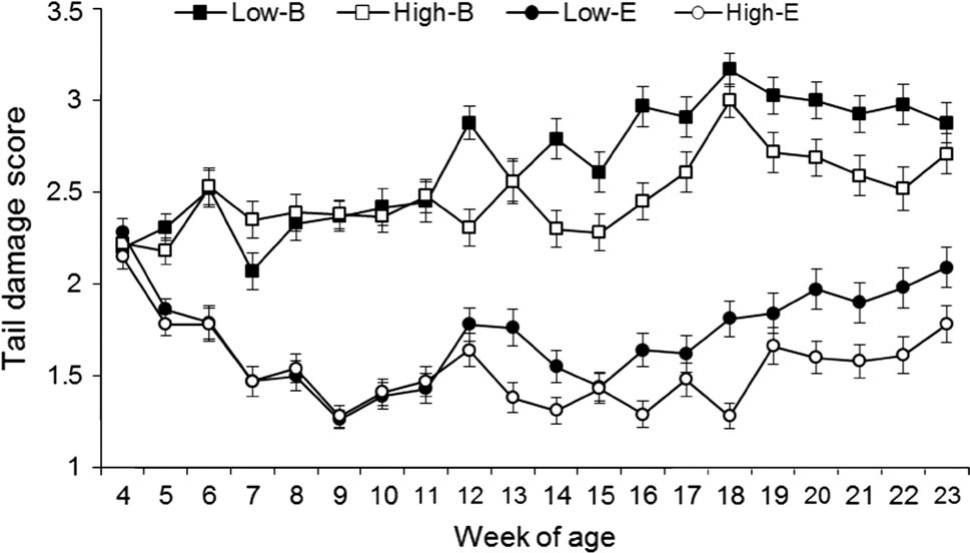
Tail damage score for high IGEg pigs in barren pens, high IGEg pigs in enriched pens, low IGEg pigs in barren pens, and low IGEg pigs in enriched pens. Note that the *y*-axis ranges from 1 to 3.5 while tail damage scores from individual pigs may range from 1 to 4

Pigs already showed tail damage from the moment of weaning, with an average tail damage score of 2.2 (Fig. 2). During the nursery phase (weeks 4–7) there was no difference between the IGEg groups for tail damage (*P* = 0.93), but a clear difference was present between barren and enriched pens (tail damage score nursery: barren 2.3 ± 0.04; enriched 1.8 ± 0.04; *P* < 0.001). During the finishing phase (weeks 8–23) high IGEg pigs had a lower tail damage score (high 2.0 ± 0.05; low 2.2 ± 0.05; *P* = 0.004), and the positive effect of enrichment remained (mean tail damage score finishing: barren 2.6 ± 0.05; enriched 1.6 ± 0.05; *P* < 0.001). This resulted in an additive effect of IGEg group and straw enrichment on tail damage, without interactions between these two factors (*P* = 0.79).

### Consumption of Jute Sacks

From week 8 onward a jute sack was attached to the wall of each pen to limit tail biting behaviour (Fig. 1). Chewing on a jute sack was indeed related to chewing on a tail, with a positive correlation between the consumption of jute sacks per pen and average tail damage on pen level (*r*_*p*_ = 0.34; *P* = 0.003). In pens with high IGEg pigs these sacks had to be replaced 30 % less often than in pens with low IGEg pigs. Over a period of 15 weeks, high IGEg pigs consumed 2.9 ± 0.3 jute sacks per pen, whereas low IGEg pigs consumed 4.2 ± 0.3 sacks per pen (*P* = 0.002). Pigs in barren pens consumed 4.3 ± 0.3 jute sacks whereas in enriched pens on average 2.8 ± 0.3 jute sacks were consumed (*P* < 0.001). There was no interaction between IGEg group and housing condition for the consumption of jute sacks (*P* = 0.84).

## Discussion

We have investigated the behavioural consequences of a single generation of divergent selection for IGEg in pigs in two housing systems. The divergent IGEg groups showed structural differences in biting behaviours directed towards pen mates and to the physical environment during the finishing phase. This indicates that selection on IGEg may alter a range of behaviours, and even behaviours not related to group members, such as biting on objects in the environment. This suggests that selection on IGEg does not merely alter social interactions, but rather results in changes in an internal state of the animal from which differences in behaviour may arise.

### Potential Underlying Mechanisms

The origin of biting behaviour may be found in amongst others aggression, frustration, stress, or maintenance of dominance relationships (Scott 1948; Marler 1976; Schrøder-Petersen and Simonsen 2001). Aggression and competition have been associated with IGEs in a wide range of taxa (reviewed by Wilson 2013), for example in laying hens (Cheng and Muir 2007), and were also expected to underlie IGEg in pigs (Rodenburg et al. 2010). Pigs selected for high IGEg did show subtle differences in aggressive behaviour (Camerlink et al. 2013), but most biting behaviour was unrelated to aggression. The expression of aggressive and competitive behaviours might, however, have been tempered by ad libitum feeding (Camerlink et al. 2014). Pigs of high IGEg were suggested to be better in establishing dominance relationships (Rodenburg et al. 2010; Canario et al. 2012; Camerlink et al. 2013), but this does not explain the differences in biting on objects.

The varying biting behaviours seem more to originate from frustration or stress. Pigs have a strong intrinsic need to root and forage, and when this need cannot find an outlet in the physical environment it may be redirected to group members (e.g. Schrøder-Petersen and Simonsen 2001). Tail biting, ear biting, and chewing on distraction material may therefore have a similar motivational background. These behaviours have also been related to frustration, stress, and fearfulness (Taylor et al. 2010; Zupan et al. 2012). Additional behavioural and physiological data suggest that high IGEg pigs may be better capable of handling stressful situations and are less fearful (Camerlink et al. 2013; Reimert et al. 2013, 2014). Similarly, laying hens selected on IGEs for survival, which is directly related to cannibalistic pecking, were less sensitive to stress and were less fearful (reviewed in Rodenburg et al. 2010). Laying hens selected for high productivity and survivability also showed neuroendocrine changes, e.g. higher dopamine and epinephrine and lower serotonin, which may underlie differences in various behaviours amongst which aggression (Cheng and Muir 2007).Tail biting and cannibalistic pecking have similar underlying needs (e.g. urge to forage, feed or explore) and causes (e.g. stress or nutritional deficiencies). Though this concerns different species, and selection for IGEs on different traits, the behavioural responses to selection have remarkable similarities which may suggest a similar mechanism in pigs and laying hens. Together, the various behaviours that are altered through selection on IGEg seem to reflect an internal state rather than solely social interactions.

### The Effect of Selection

In this study, many behaviours have been tested for statistical significance, which increases the risk of false positives due to chance. However, we found a systematic pattern of less biting behaviour in high IGEg pigs, which was supported by small *P* values that are unlikely to be chance results. We believe that the four significant results all relating to biting behaviour, with an average *P* value of ~0.005 (biting, chewing toy, jute sacks consumed, and tail damage score) indicate a true effect. Behavioural effects may appear after only few generations of selection, as for example seen in laying hens selected based on direct and indirect genetic effects (Bolhuis et al. 2009). We did not observe differences between IGEg groups in tail biting behaviour itself, which might be due to the scan sampling method, whereby infrequent short lasting behaviours are easily missed (Altmann 1974). Tail biting behaviour and the emergence of a small wound on the tail may initially occur unnoticed (Ursinus et al. 2014), and it should be emphasized that tail damage in the current study regarded bite marks rather than wounds. The higher tail damage score in low IGEg pigs indicates that low IGEg pigs did spent more time on tail biting or were biting more fiercely. Biting behaviour, and especially tail biting, is considered an important animal welfare issue and our results seem to confirm the hypothesis that selection on IGEg may contribute to a solution (D’Eath et al. 2014).

The potential effect of IGEg on harmful biting behaviour might have been underestimated in the current trial. The circumstances of the trial were more favourable compared to common (Dutch) intensive farming conditions (more space per animal), and control measures were taken to limit tail biting (daily treatment of wounded tails, provision of wood shavings and jute sacks, and the removal of animals with shortened tails). In particular, part of the disposition to bite may have been redirected to chewing on the jute sack (Fraser et al. 1991; Van de Weerd and Day 2009). This together may have reduced tail biting and may have prevented a severe outbreak (Zonderland et al. 2008; Statham et al. 2009). Interference in possible underlying mechanisms of IGEs, for example changing resource availability, might alter the effect of selection (Arango et al. 2005; Wilson 2013). With no interference in the cannibalistic pecking of laying hens, clear differences between high and low IGE selection lines were found (reviewed in Rodenburg et al. 2010). From a scientific perspective, measures to limit tail biting would ideally have been omitted, but this would go against ethical regulations of animal experiments. If biting behaviour would be one of the mechanisms underlying IGEg in pigs, then control measures may have reduced the expression and effect of selection.

It was also suggested that selection on behaviour or IGEg might alter activity (D’Eath et al. 2010; Rodenburg et al. 2010), whereby the positive effect on the growth rate of others would occur due to apathy of the animal, resulting in a reduced number of social interactions, and thus also a reduced negative impact on the growth rate of others. The activity level of high and low IGEg pigs did not differ in the current study, which suggests no such response to selection.

### Considerations for Implementation

Previously, behavioural changes were suggested in a small experiment applying selection on IGEg in pigs (Rodenburg et al. 2010). Behavioural differences were also noted in a multiple-generation selection experiment based on the performance of groups of half sibs, thus including direct and indirect genetic effects (Gunsett 2005). The current study is, however, the first large scale experiment evaluating the behavioural consequences of selection on IGEg in a large mammal. Knowledge on the mechanisms behind IGEg in pigs may contribute to the optimization of pig breeding and farming. For example, insight in which inherited behaviours affect growth rate of group mates may outline the potential possibilities, and potential profitability, of reducing or enhancing specific (social) interactions. Tail biting may hereby be a factor, as tail biting has found to be heritable in some breeds (Breuer et al. 2005) and victims of tail biting may show a reduced growth rate (e.g. Sinisalo et al. 2012). Follow-up research under commercial conditions, and selection over multiple generations, would be essential to gain further insight in the magnitude and potential variability of the behavioural and physiological changes on the long term. If selection on high IGEg causes pigs to show less harmful biting behaviour, then over generations, other behaviours might emerge in relation to IGEg.

### Benefits from Both Genetics and Environment

G × E interactions exist for pig production traits (Schinckel et al. 1999), but are to date not shown for behaviour in finishing pigs (e.g. Guy et al. 2002). Little G × E interactions for pig behaviour were found in the current study, and it is therefore not expected that genetic selection on IGEg would alter behaviour differently in different housing conditions. Provision of straw resulted in more behaviour directed towards the environment, which is in accordance with literature (e.g. Fraser et al. 1991). The reduction in damaging behaviour and the lower tail damage scores of pigs on straw clearly point out the potential of substrate to improve pig health and welfare. Tail damage was further reduced in pigs selected for high IGEg, which suggests that differences in the genetic disposition to perform tail biting remain present also when suitable substrate is provided. This shows that biting behaviour can be reduced from two approaches, namely by redirecting the biting behaviour towards the environment instead of conspecifics through the provision of suitable substrate, and by reducing the motivation to bite through selection on IGEg. Straw is often regarded the most suitable substrate to reduce tail biting (Zonderland et al. 2008; Van de Weerd and Day 2009), but selection on IGEg may give an additional reduction that is cumulative over generations, leading to a further increase in animal welfare.

## Conclusion

Selection on high IGE for growth in pigs reduced biting behaviour, which was expressed in lower occurrences of aggressive biting, ear biting, biting on materials provided for chewing (including jute sacks), and less tail damage due to tail biting. The availability of straw in the pen reduced the expression of pen-mate directed behaviours. Hereby straw may redirect the biting behaviour to the environment, whereas selection for IGEg may reduce the disposition to bite. Both may therefore lead to improvements in animal welfare. We outlined some aspects for further research and would like to emphasize that the impact of selection for IGEs for production traits may reach further than solely social interactions.

## Appendix

Refer Table 3.

**Table 3:** Ethogram

Behaviour	Description
General individual	
Sleeping	Lying without performing any other described behaviour, eyes closed
Lying inactive	Lying without performing any other described behaviour, eyes opened
Standing	Standing without performing any other described behaviour
Locomotion	Walking or running without performing any other described behaviour
Sitting	Sitting or kneeling without performing any other described behaviour
Comfort behaviour	Rubbing body against objects or pen mate, scratching body with hind leg or stretching (part of) body
Eating feeder	Eating at feeder
Drinking	Drinking from drinking nipple
Urinate/defecate	Urinating or defecating
Exploration	
Exploration floor	Sniffing, touching or scraping floor
Nosing object	Nosing above floor level
Rooting	Rooting pen floor or in wood shavings or straw
Rooting object	Rooting above floor level or object
Chewing	Non-feed chewing or chewing straw
Chewing toy	Chewing toy: chain with ball or jute sack
Social	
Nosing head or body	Touching/sniffing any part of a pen mate except nose
Nose contact	Mutual nose contact
Playing	Individual or group wise gamboling, pivoting: running around the pen, sometimes with gently nudging of pen mates
Belly nosing	Rubbing belly of a pen mate with up and down snout movements
Mounting	Standing on hind legs while having front legs on other pig’s body
Aggression	
Fighting	Ramming or pushing a pen mate with or without biting the pen mate. Can be either mutual or individual
Head knocking	Head knock given at place other than feeder
Biting	Bite given at other place than feeder
Fighting at feeder	Push, head knock or bite given at feeder
Oral manipulation of group mates	
Tail biting	Nibbling, sucking or chewing the tail of a pen mate
Ear biting	Nibbling, sucking or chewing the ear of a pen mate
Manipulating other	Nibbling, sucking or chewing part of the body of a pen mate
